# Feature evaluation of accelerometry signals for cough detection

**DOI:** 10.3389/fdgth.2024.1368574

**Published:** 2024-03-22

**Authors:** Maha S. Diab, Esther Rodriguez-Villegas

**Affiliations:** Wearable Technologies Lab, Department of Electrical and Electronic Engineering, Imperial College London, London, United Kingdom

**Keywords:** accelerometer, cough, cough detection, feature extraction, feature evaluation, respiratory diseases, time-domain features, wearables

## Abstract

Cough is a common symptom of multiple respiratory diseases, such as asthma and chronic obstructive pulmonary disorder. Various research works targeted cough detection as a means for continuous monitoring of these respiratory health conditions. This has been mainly achieved using sophisticated machine learning or deep learning algorithms fed with audio recordings. In this work, we explore the use of an alternative detection method, since audio can generate privacy and security concerns related to the use of always-on microphones. This study proposes the use of a non-contact tri-axial accelerometer for motion detection to differentiate between cough and non-cough events/movements. A total of 43 time-domain features were extracted from the acquired tri-axial accelerometry signals. These features were evaluated and ranked for their importance using six methods with adjustable conditions, resulting in a total of 11 feature rankings. The ranking methods included model-based feature importance algorithms, first principal component, leave-one-out, permutation, and recursive features elimination (RFE). The ranking results were further used in the feature selection of the top 10, 20, and 30 for use in cough detection. A total of 68 classification models using a simple logistic regression classifier are reported, using two approaches for data splitting: subject-record-split and leave-one-subject-out (LOSO). The best-performing model out of the 34 using subject-record-split obtained an accuracy of 92.20%, sensitivity of 90.87%, specificity of 93.52%, and F1 score of 92.09% using only 20 features selected by the RFE method. The best-performing model out of the 34 using LOSO obtained an accuracy of 89.57%, sensitivity of 85.71%, specificity of 93.43%, and F1 score of 88.72% using only 10 features selected by the RFE method. These results demonstrate the ability for future implementation of a motion-based wearable cough detector.

## 1 Introduction

Chronic Respiratory Diseases (CRDs) are common health conditions that affect the respiratory tract airways and lungs, resulting in various symptoms such as dyspnea (difficulty breathing), chest pain, cough, sputum, and others. Some diseases that fall under the CRD umbrella are chronic obstructive pulmonary disease (COPD), asthma, pneumonoconiosis, interstitial lung disease, and pulmonary sarcoidosis. These are incurable yet treatable conditions that require continuous monitoring of the disease’s progression and an adjustable treatment plan. Among those conditions, the two main contributors to the global burden of chronic respiratory diseases are asthma and COPD (1), with the latter being the third cause of death worldwide (2). Moreover, global health metrics in 2019 concluded that CRDs were responsible for 71.31 million years of life lost (YLLs) and 32.4 million years of healthy life lost due to disability (YLDs) (1).

To help ease this global health burden inflicted by CRDs and to limit patients’ deterioration, possible solutions include early detection and continuous monitoring of the disease to allow for proper treatment plans and on-time interventions when needed. To achieve this, many researchers targeted the detection and monitoring of respiratory diseases by leveraging the potential of data-driven artificial intelligence (AI) algorithms. The studies focused on the use of medical symptoms as an indicator to the presence of a respiratory health problem—detection— and as a measure of the progression of the respiratory condition—monitoring. Some of the symptoms that have been analyzed and studied include respiratory sounds produced due to illness, such as wheeze, stridor, crackle, and gasp (3), and the symptom that has been the center of attention most recently is coughs.

The use of sophisticated machine learning (ML) and deep learning (DL) algorithms for the detection and the diagnosis of respiratory diseases from cough sounds has become a research focus in the last few years. Researchers have employed linear ML methods such as linear regression (LR) (4–6), non-linear ML methods including both support vector machine (SVM) (7–9) and k-nearest neighbors (kNN) (10–12), ensemble methods such as random forest (RF) (13, 14), and XGBoost (15), and more complex DL methods such as artificial neural network (ANN) (16) and convolutional neural network (CNN) (17, 18) to detect cough sounds. These studies targeted the use of audio recordings to detect the presence of cough events achieving an accuracy or sensitivity above 90%. Similar algorithms were also used for the diagnosis of different respiratory conditions, with more recent studies focusing on the diagnosis of COVID-19 from audio recordings such as the case in Imran et al. and Wei et al. (19, 20) using CNN models to differentiate between COVID-19, pertussis, and bronchitis. In addition, these advances in cough detection and diagnosis algorithms were then taken a step further to provide a portable, ubiquitous solution by implementing them within a mobile App. This provided a cost-effective, easy-to-use solution that witnessed a rise in its use especially during the COVID-19 pandemic. Some of these mobile Apps are COVID-19 sounds app (21) used for data collection, AI4COVID-19 (19), and QUCoughScope (22) for both data collection and diagnosis.

Though the use of the conventional audio-based method for cough detection and diagnosis has resulted in acceptable performance, it is dependent on capturing the audio during a cough event. This requires the use of always-on microphones that are always recording/listening to conversations and personal information in the wait for a cough event. As such, the dependency on audio for cough detection presents both privacy and security issues where private conversations could be maliciously used. In addition to the privacy invasion, another disadvantage of this portable solution is phone battery depletion, since the App has to be working in the background at all times. Therefore, an alternative solution to the audio-based method is needed for the implementation of a portable wearable cough detector.

In this work, we investigate the use of motion-based detection in place of the audio-based detection method. The rationale behind this is explained by the fact that the audible cough sound that is commonly used for detection is the result of a physical motor pattern of the body. The forced expulsive pattern of cough can be broken down into three phases: (i) inspiratory phase— characterized by typical inspiration (drawing of air into lungs); (ii) compression phase—glottis is completely closed and thorax is compressed to increase subglottic pressure; (iii) expulsive phase—glottis opens rapidly resulting in high-pressure airflow (23, 24). These motions leading to a cough can be captured using an accelerometer worn close to the body, allowing the collection of data related to coughing.

Accelerometer sensors have been mostly used in applications related to human activity recognition (HAR) such as fitness tracking, with limited research targeting the use of an accelerometer as a cough sensor. One of the few studies using motion-based cough detection was presented in Mohammadi et al. (25) using a dual-axis contact accelerometer placed at the patient’s neck to capture epidermal vibrations. A total of 35 features composed of temporal, time–frequency, frequency, and information-theoretic were extracted from the accelerometry signals. A binary genetic algorithm (BGA) was used for feature selection together with an SVM model to differentiate between voluntary cough and rest, achieving an accuracy of 99.26±0.12%. Other works targeting the use of only accelerometry signals were presented throughout multiple works (26–28) comparing the performance of cough detection in accordance to the position of the sensor. A total of five different positions were examined: chest, stomach, arm—using a tri-axial accelerometer attached by an elastic belt, ear using a headset with a fastened accelerometer on one side, and finally shirt pocket using a tri-axial accelerometer of the phone placed freely and firmly in the pocket. Raw data of the x, y, and z axes were used to train a CNN model to detect cough events. The best performance was achieved at the ear and chest positions with an accuracy of 97% for both (26). Further development used spectral summation of the tri-axial signal together with a CNN model to boost the performance demonstrating up to approximately 99% accuracy in the ear position and 98.2% accuracy at the chest (27). A different approach was used in Vyas and Doddabasappla (28), where spectrum spread of only the y and z axes were used as input to kNN and SVM models comparing only three positions (chest, stomach, and pocket). The SVM model outperformed the kNN model in detecting cough events, with the best accuracy being achieved in the pocket position at 96.1%, followed by the chest at 94.3%, and then the stomach at 94%. Other studies that are worth mentioning have used an accelerometer together with other sensors, such as in Otoshi et al. (29), that used a strain sensor around the neck with a tri-axial accelerometer around the epigastric region. Another study used a tri-axial accelerometer with an ECG front-end placed around the chest for cough detection (30).

In the reviewed cases of accelerometer-based cough detection, the used accelerometer was a contact sensor that required direct attachment to the user’s skin either around the neck, chest, stomach, or ear. The only case with a non-contact accelerometer was the use of a phone placed freely in a shirt’s pocket. In both cases, considering the end-user’s need for continuous monitoring, the use of a contact-based sensor is most likely to present discomfort in the long run, while the use of a phone or other non-contact accelerometer with free placement in the pocket is not practical and is prone to error due to inconsistent positioning and added noise from the movement of the sensor within the pocket.

This leads to the main purpose behind this work: to the authors’ best knowledge, there have not been any evaluation studies of time-domain features extracted from non-contact accelerometry signals for motion-based cough detection. Therefore, the first objective of this research study—an extension of our previous studies (31, 32)—is investigating the use of a non-contact accelerometer for motion-based cough detection using data collected from multiple subjects. The second objective is to evaluate the ability of time-domain features to distinguish between cough and non-cough movements. This focus on time-domain features is because the extraction of these features does not require the additional step of computing the signal’s frequency components as the case is for frequency-domain features. Hence, the computation of time-domain features requires less computational time and space/memory. The third objective of the study is to compare different feature importance methods used for feature selection and use their feature ranking results to select the top features. Finally, the fourth objective of the study is to build a simple binary classification model for motion-based cough detection using different number of selected features based on different ranking methods, to find the best model with optimum performance and number of features.

The rest of the paper is organized as follows: the materials used for data acquisition and methods for pre-processing and data preparation are covered in Section 2, together with the description of the extracted time-domain features and the feature evaluation methods used. The data distribution of the extracted features and the results of the feature importance ranking are presented in Section 3, as well as the performance metrics used and the results of the classification models for cough detection. Finally, the results are discussed, and the different models’ performances are compared, based on the number of features and the type of ranking method used, in Section 4, with some concluding remarks.

## 2 Materials and methods

To correctly classify and differentiate between cough and non-cough samples from accelerometry signals, a set of features was extracted for evaluation and comparison. The computed features were chosen based on the results of the literature review of related accelerometer-based activity recognition research, with a focus on time-domain features. The reason behind these targeted time-domain features is to evaluate and investigate the feasibility of cough detection based on only time-domain extracted features, which require a minimum amount of time and space for computation compared to frequency-domain features. In this section, the methods used for data collection, pre-processing, feature extraction, and feature evaluation are discussed.

### 2.1 Data collection

A total of five subjects (three males and two females) with an average age of 27.6±3.1 years participated in the experiment. The study was approved by the Imperial College Research Ethics Committee (ICREC ref.: 6669400), and written informed consent was obtained from all subjects. The subjects had a Nordic Thingy:53—a multi-sensor IoT prototyping platform—clipped to their shirt’s collar. Accelerometry signals were recorded while subjects were seated for both cough and non-cough events, resulting in a total of 180 recordings (90 cough and 90 non-cough) each of 10 s duration, at a sampling frequency of 62.5 Hz. Four subjects recorded 20 cough sessions and 20 non-cough sessions, while one subject recorded 10 cough sessions and 10 non-cough sessions. The cough sessions required subjects to cough throughout the 10 s recording continuously and repeatedly. Non-cough sessions involved the subject resting, talking, and/or using a mobile phone.

### 2.2 Data pre-processing

The acquired accelerometer recordings were composed of tri-axial signals in the x-axis, y-axis, and z-axis indicating sideways movement (right–left), vertical movement (up–down), and outward movement (forward–backward), respectively. The first step in the pre-processing stage was the computation of the accelerometer vector magnitude by combining the raw x, y, and z accelerometry signals using (1). The magnitude vector describes the overall movement and is commonly used in processing accelerometer data. After the computation of the magnitude, each recording became composed of four vectors (accX,accY,accZ,Mag), which were then filtered using a fourth-order Butterworth Bandpass filter (0.5–15 Hz) to filter out movements below 0.5 Hz and any DC offset; and to eliminate the frequencies above 15 Hz, where no peaks were observed in cough recordings.

(1) $$Mag=\sqrt{x^{2}+y^{2}+z^{2}}$$

**Figure 1:**
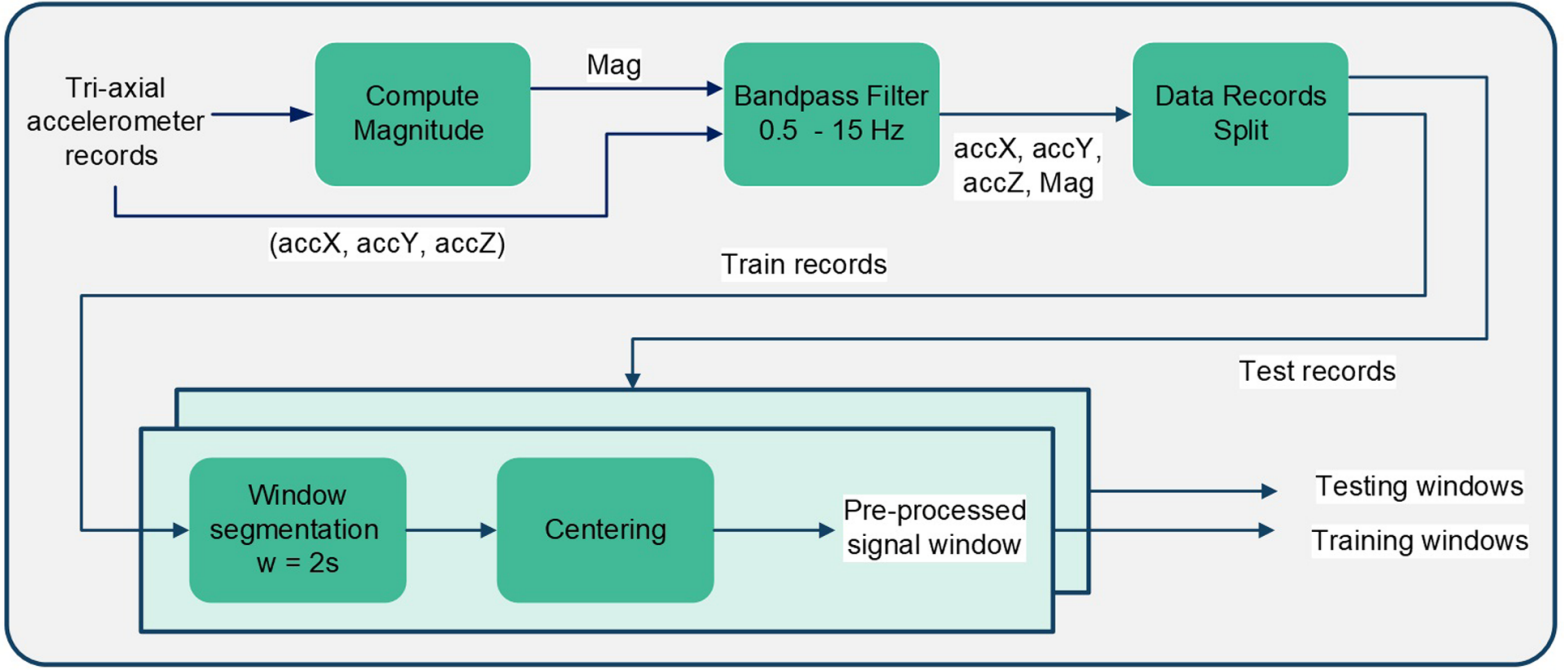
Block diagram summarizing the pre-processing steps implemented on the acquired tri-axial accelerometry signals.

**Figure 2:**
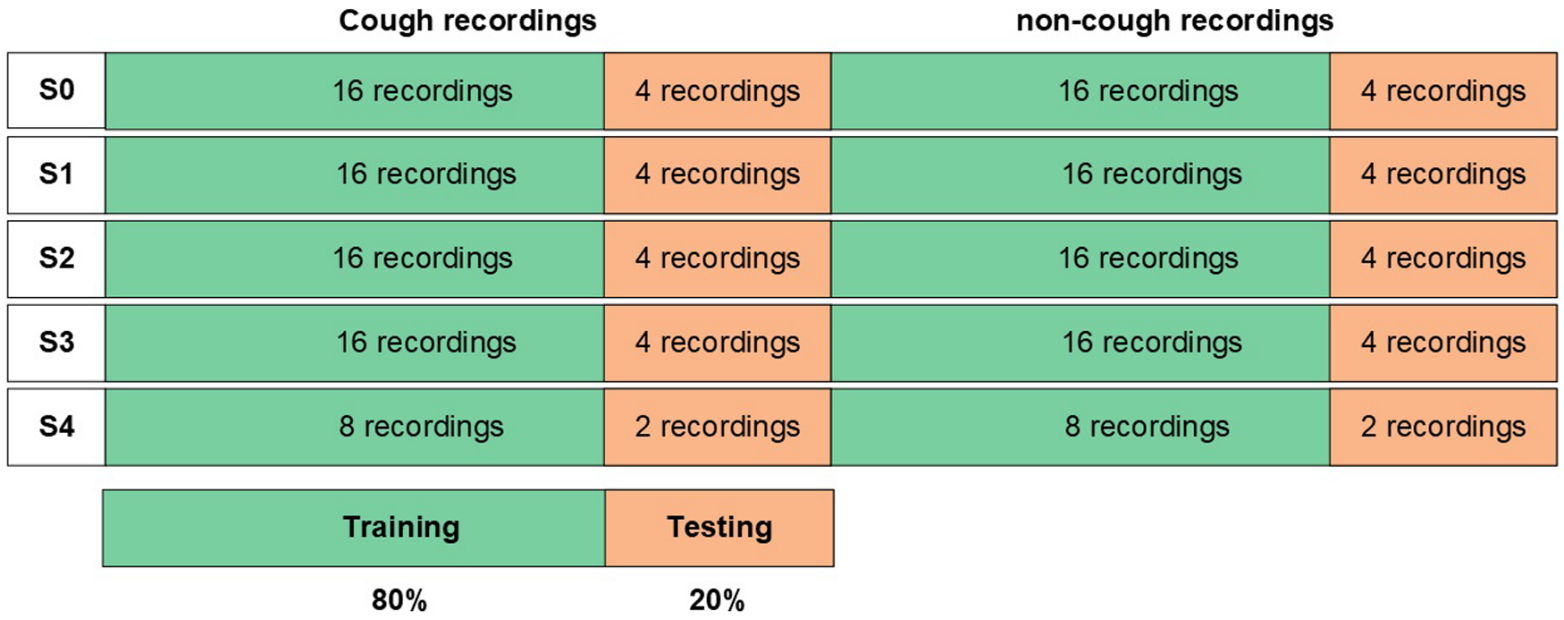
Detailed process for the data partitioning visualized in Figure 1 under the “Data Records Split” block for cough detection.

The implemented pre-processing pipeline is summarized in Figure 1, where after filtration the data were split into training and testing. Records were split based on subjects, where 80% of records from each subject were kept for training and the remaining 20% recordings from the same subject were kept for testing, with a balanced division between cough and non-cough recordings. The division of recordings is visualized in Figure 2. Afterward, each recording was segmented into windows of size 2s and a sliding window of 200ms resulting in a total of 42 segments/samples per recording. Window segmentation of training and testing records resulted in a total of 6,048 balanced training windows and 1,512 balanced testing windows. Following record segmentation, each window—composed of four signals—was centered by subtracting the mean of the corresponding signal vector as given in Equation 2, where $\omega^{\prime }$ is the raw signal window, ω is the resultant centered window, and i corresponds to the accelerometer signal vector within a window (i=accX,accY,accZ,Mag).

(2) $$\omega_{\left(i\right)}=\omega_{\left(i\right)}^{\prime }-\bar{\omega_{\left(i\right)}^{\prime }}$$

### 2.3 Feature extraction

**Figure 3:**
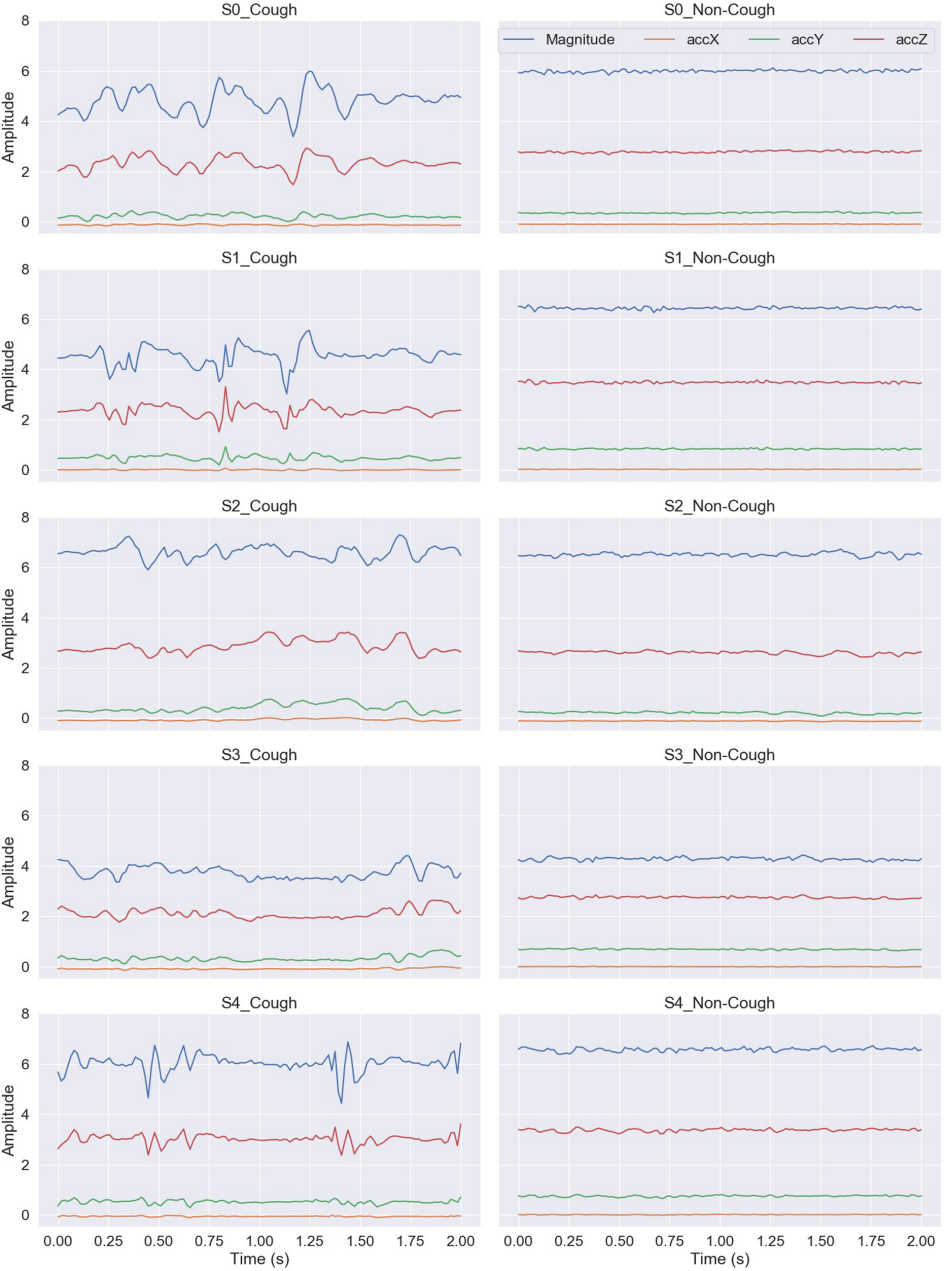
An example illustrating a cough and a non-cough 2 s window of the captured accelerometry signal from each of the five subjects.

The audible cough sound made by a subject is the result of a chest motor pattern that is captured by the acquired accelerometer recordings. An example of the acquired accelerometry signal for a cough and a non-cough window (before centering) from each of the five subjects is presented in Figure 3. The cough accelerometer signal displays a non-periodic, random behavior that can be used, once detected, to differentiate it from the less random non-cough accelerometry signals. Hence, time-domain features measuring the distribution, dispersion, and randomness of the signal were computed to distinguish between cough and non-cough signals.

Following the pre-processing stage, the time-domain features were extracted from the centered windows. A set of statistical features was computed from each of the signals (accX,accY,accZ,Mag) to describe the captured movement during cough and non-cough events. All computations were performed in Python using pandas, scipy.stats, and EntropyHub libraries. The list of the time-domain features used is summarized in Table 1 and a brief description is given as follows:

**Minimum** (Min) is the minimum amplitude value of the accelerometer signal within a window.
**Maximum** (Max) is the maximum amplitude value of the signal within a window.
**Difference** (Diff) is the amplitude range calculated by subtracting the minimum value from the maximum value of the signal within a window.
**Root mean square** (RMS) is the square root of the arithmetic mean of the squared values of the signal within a window. The RMS feature can evaluate the signal amplitude and energy in the time domain. For our centered windows (mean = 0), the RMS is also equal to the standard deviation, hence only RMS as a feature was computed.
**Variance** (Var) is the measure of the data dispersion of the accelerometer signal within a window from the mean, which is also the standard deviation squared.
**Interquartile range** (IQR) is the measure of dispersion and spread of the accelerometer signal within a window, computed by the difference between the 75th and the 25th percentile. It is similar to standard deviation and variance in measuring the dispersion of the signal; however, it is more robust against outliers.
**Median absolute deviation** (MAD) is another measure of the data dispersion that is also more robust against outliers. It computes the median of the absolute deviation from the median of the signal within a window.
**Skewness** is the measure of the symmetry of the distribution of the accelerometer signal within a window. A normal distribution has a skewness of 0, while a negative skewed distribution indicates a distribution with the tail in the negative side, and a positive skewed distribution has a tail in the positive side.
**Kurtosis** is another measure for the distribution shape of the signal within a window describing the peak of the distribution. Using Fisher’s definition of kurtosis—the normal distribution of kurtosis is 0, if the value is less than 0 the distribution has a flatter shape, while if greater the distribution has a sharper shape.
**Entropy** (Ent) is the mean of the computed approximate entropy of the signal within a window. It is used to measure the signal randomness and disorder.
**Correlation** (Corr) is the measure of the pairwise correlation between the tri-axial signals within a window: Corr(accX and accY), Corr(accY and accZ), and Corr(accX and accZ).

**Table 1:** List of extracted features for evaluation.

Feature type	Size	Feature names
Minimum	4	Min_X , Min_Y , Min_Z , Min_Mag
Maximum	4	Max_X , Max_Y , Max_Z , Max_Mag
Difference	4	Diff_X , Diff_Y , Diff_Z , Diff_Mag
RMS	4	RMS_X , RMS_Y , RMS_Z , RMS_Mag
Skewness	4	Skewness_X , Skewness_Y , Skewness_Z , Skewness_Mag
Kurtosis	4	Kurtosis_X , Kurtosis_Y , Kurtosis_Z , Kurtosis_Mag
IQR	4	IQR_X , IQR_Y , IQR_Z , IQR_Mag
Variance	4	Var_X , Var_Y , Var_Z , Var_Mag
Entropy	4	Ent_X , Ent_Y , Ent_Z , Ent_Mag
MAD	4	MAD_X , MAD_Y , MAD_Z , MAD_Mag
Correlation	3	Corr_xy , Corr_yz , Corr_xz
Total features	43	

### 2.4 Feature importance scoring methods

With the extraction of a total of 43 features from the accelerometry signals, the importance and contribution of individual features toward cough classification must be evaluated. This evaluation ranks the features based on their importance, which later guides the process of feature selection for the final classification model. There are various feature selection methods found in the literature that can be used to rank the feature’s importance. In this study, we implemented a total of 11 feature ranking methods using only the training windows. The implemented methods and the description of their feature evaluation approaches are as follows:

#### 2.4.1 Model-based feature importance

Model-based feature importance method is dependent and specific to the machine learning algorithm used. Under this method, three tree-based models were used to rank the feature importance using the built-in importance function of the following models: XGBoost, decision tree (DT), and random forest. The XGBoost model computes feature importance based on “gain,” which is the improvement in the model’s accuracy when a feature is added to the branch. While both DT and RF models compute importance score based on the reduction of the Gini impurity, where Gini is the criterion used to evaluate the quality of a split.

#### 2.4.2 Principle component analysis

Principal component analysis (PCA) is a common method used for dimensionality reduction rather than feature selection. However, the first principal component (PC1) returned by the analysis can be used to score the importance of features. The rationale behind this is that plotting the cumulative explained variance for each component produced by our training data shows that the first component (PC1)—using a single feature, can explain over 80% of the variance in the dataset. Hence, the values of PC1 loadings were used to rank the features based on the correlation between the features and the principal component.

#### 2.4.3 Leave-one-out

As the name suggests, this method iteratively leaves one feature out at a time and evaluates the effect of its removal on the model’s accuracy. The importance was measured as the difference between the base accuracy and the new accuracy without the feature; the greater decrease in the model’s accuracy reflected the weight and importance of a given feature. Thus, higher importance and ranking were given to features with a greater decrease in the model’s accuracy when eliminated.

#### 2.4.4 Permutation

In the permutation importance method, feature ranking was based on the effect a feature had on the model’s performance after having its values randomly shuffled. Once a feature was shuffled, the new accuracy was measured and compared to the baseline accuracy; this process was repeated 10 times for each feature and the mean value was measured. The greater the drop in accuracy indicated a higher ranking of the feature. This method was implemented using the permutation_importance function in the sklearn.inspection library, with 10 repetitions for each feature permutation.

#### 2.4.5 Recursive feature elimination

In this method, the feature selection function was given a fitted trained model to evaluate and rank features by recursively considering a smaller set of features. This process of feature pruning was repeated until the number of required features was reached. In this study, the method was repeated for four different numbers of selected features, setting the desired number of features to 10, 20, 30, and, finally, 43, i.e., using all features.

#### 2.4.6 Correlation

The pairwise correlation was measured between the individual features and the binary target (cough, non-cough). Having both numerical and categorical variables, Spearman’s correlation method was computed, and the absolute value was used to rank the feature importance.

## 3 Results

**Figure 4:**
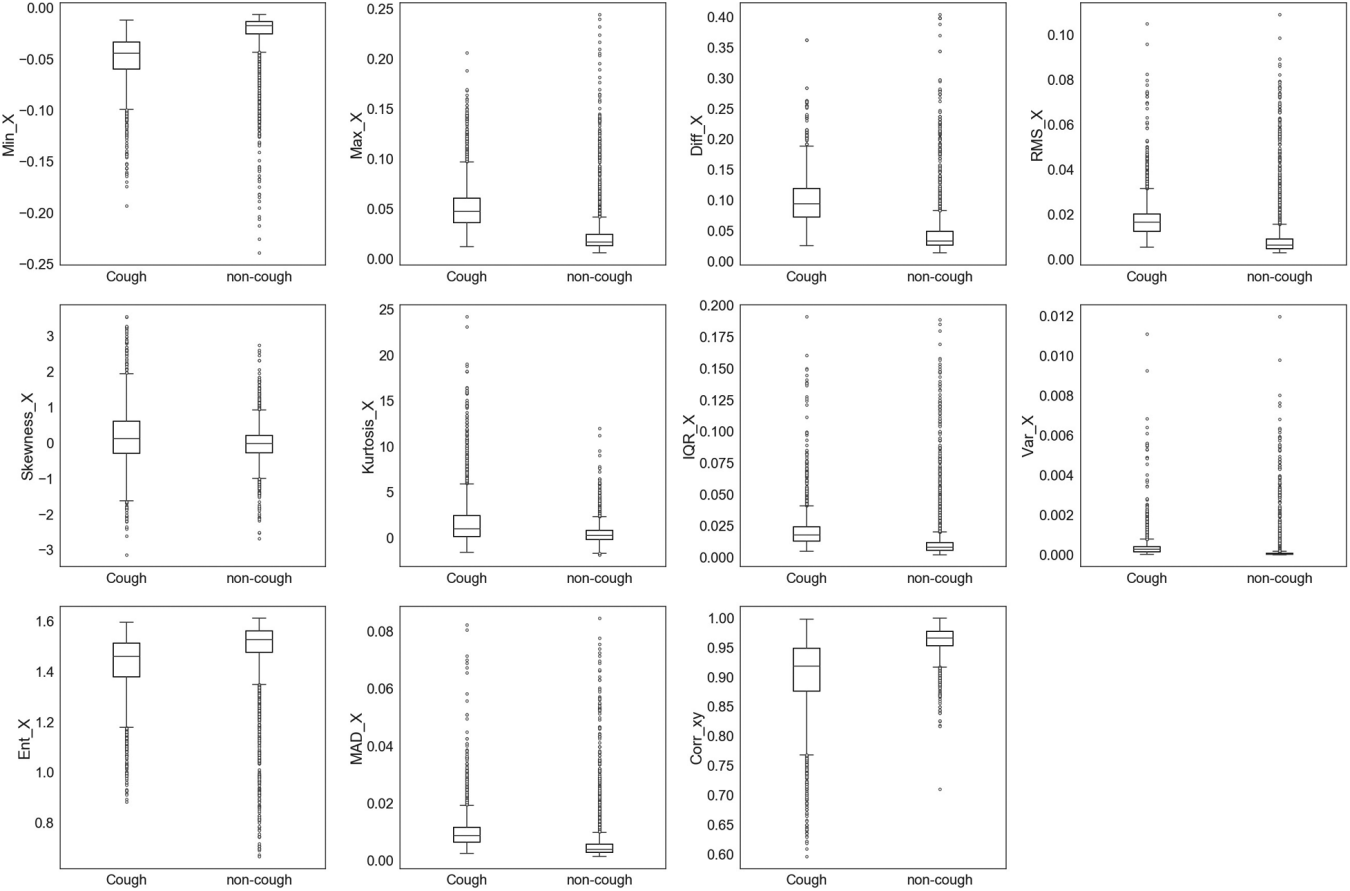
Box and whisker plot summarizing the distribution of data for 11 extracted features from the accelerometer x-axis.

**Figure 5:**
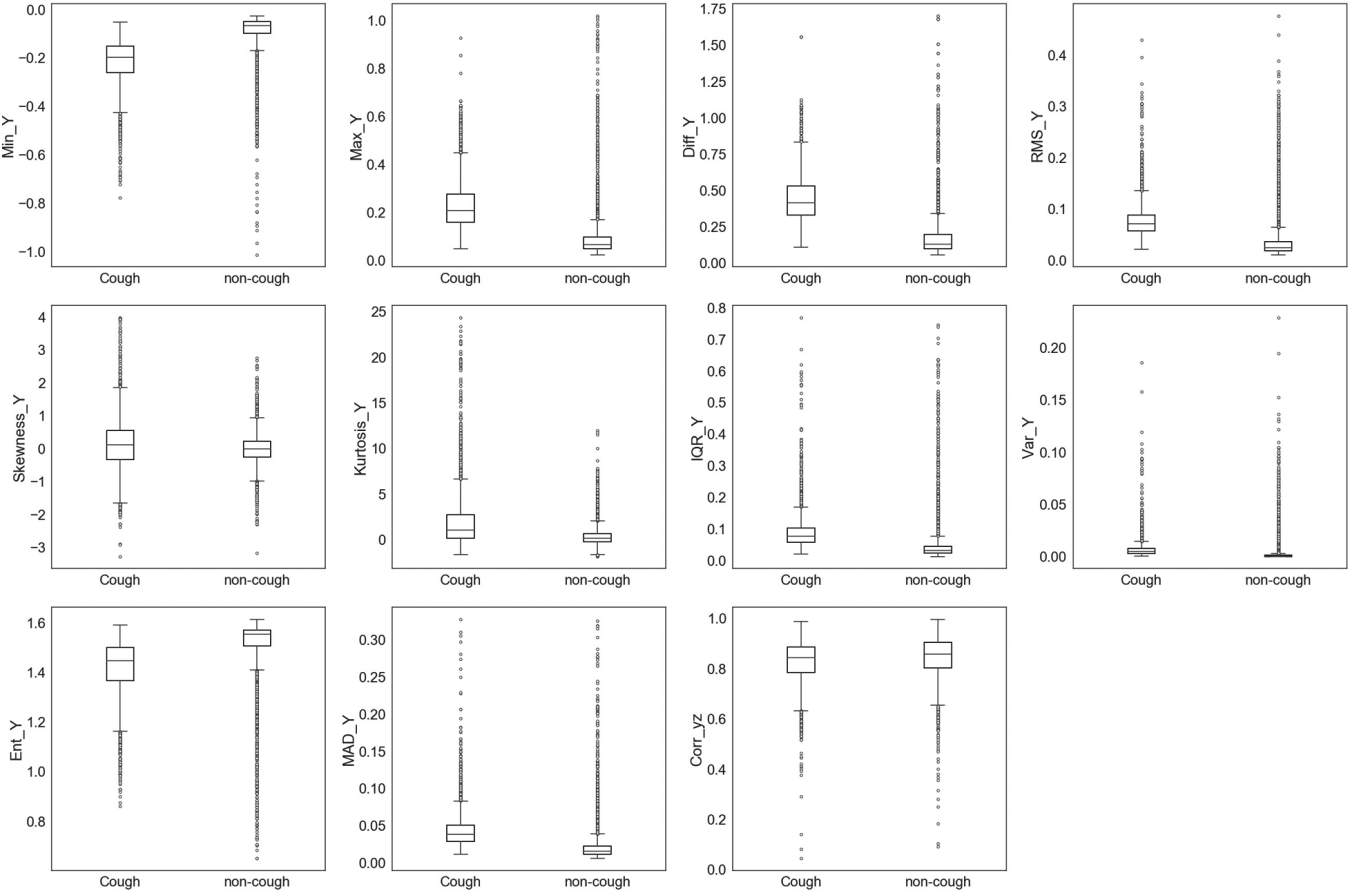
Box and whisker plot summarizing the distribution of data for 11 extracted features from the accelerometer y-axis.

**Figure 6:**
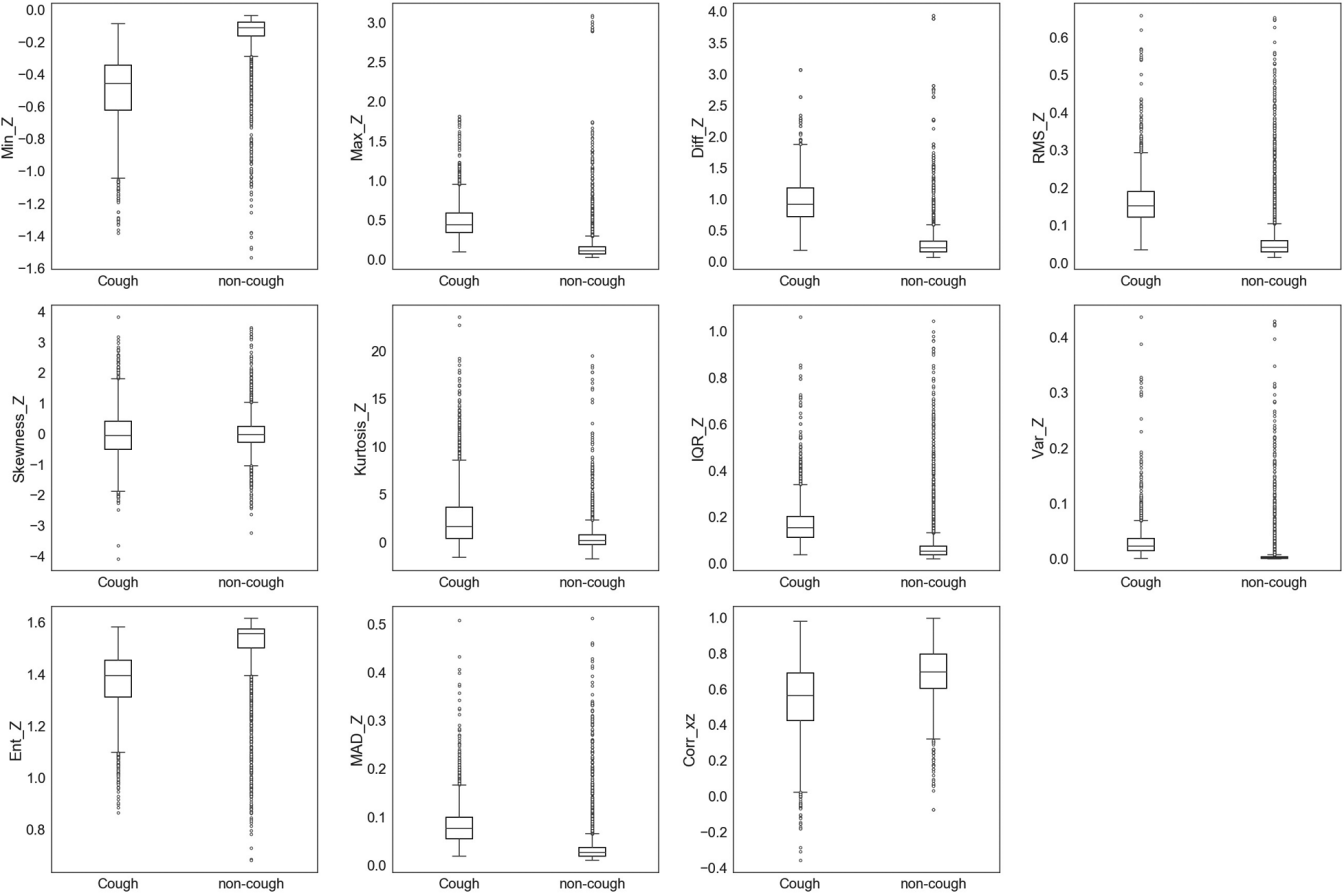
Box and whisker plot summarizing the distribution of data for 11 extracted features from the accelerometer z-axis.

**Figure 7:**
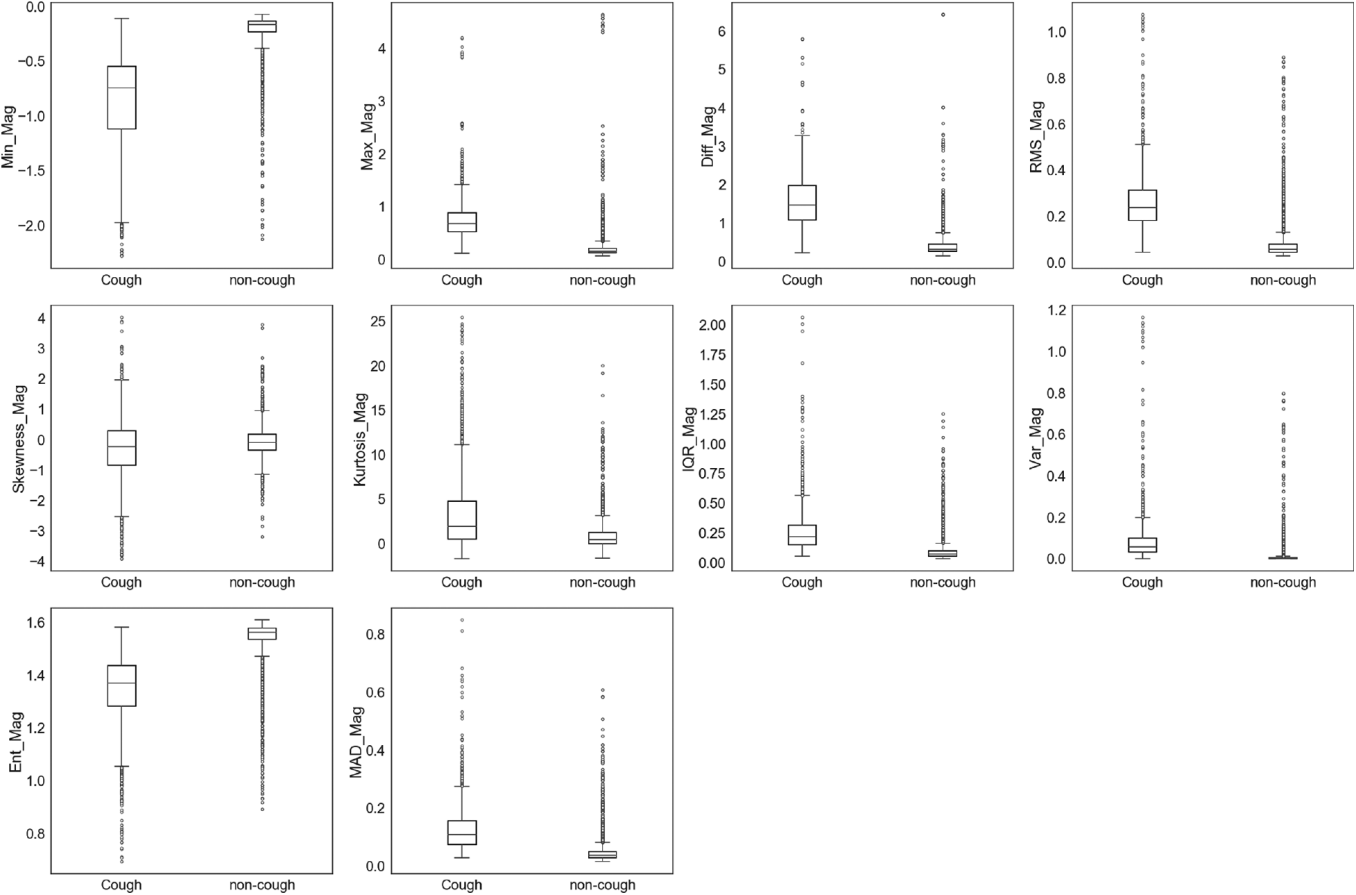
Box and whisker plot summarizing the distribution of data for 10 extracted features from the accelerometer magnitude.

The extracted features were initially assessed for their ability to uniquely identify and separate cough events from non-cough events. This assessment was performed by inspecting individual feature’s distribution based on target class (cough, non-cough). Box and whisker plots were chosen for the visual summary of a feature’s distribution. Figures 4–7 present the 43 features box and whisker plots. Features extracted from the accelerometer x-axis are given in Figure 4, accelerometer y-axis in Figure 5, accelerometer z-axis in Figure 6, and accelerometer magnitude in Figure 7. It is clear that some features can distinguish between both classes, while other features have some overlap but can still be used to separate the two categories. In a few cases, the features have very close distribution indicating their redundancy for the classification problem.

**Figure 8:**
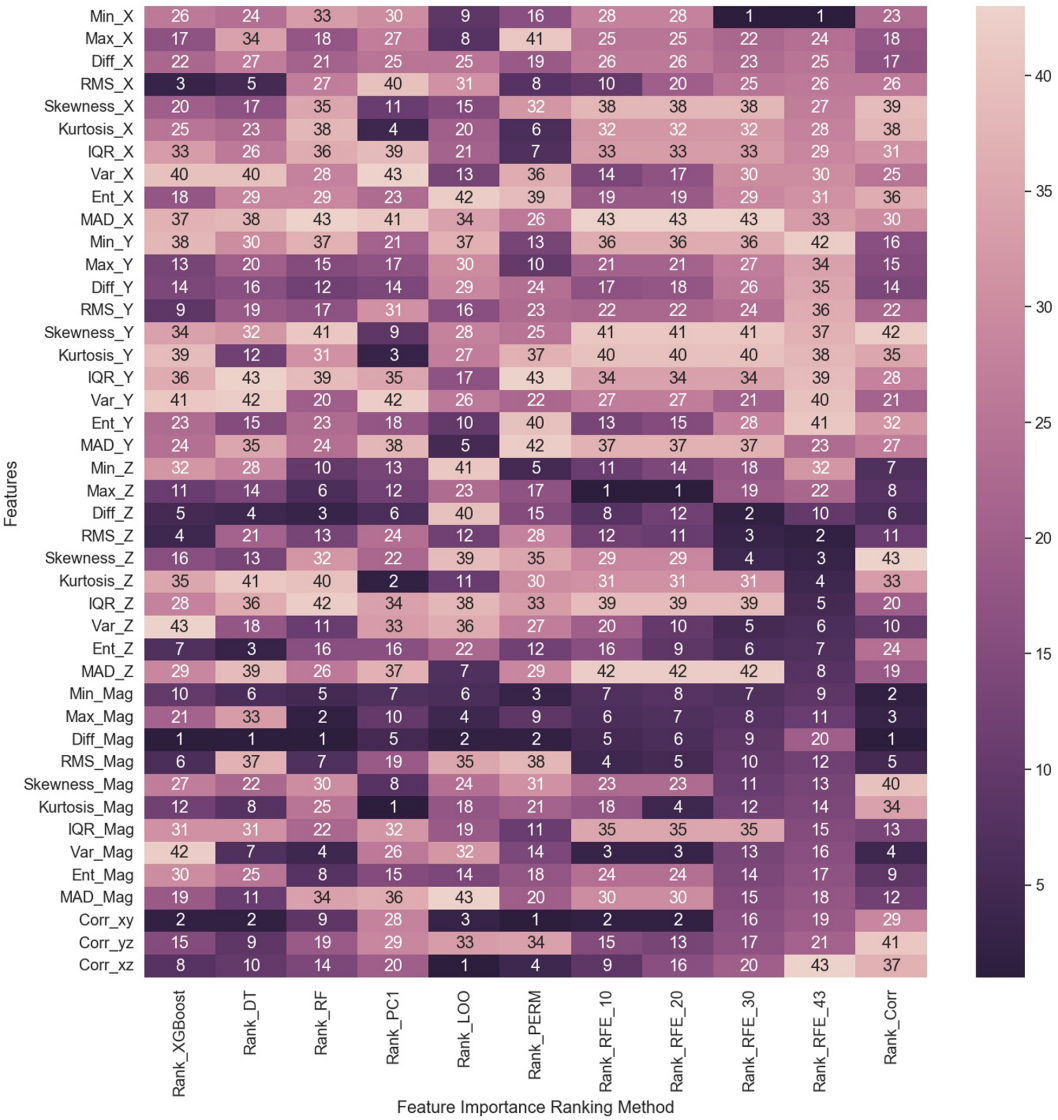
Heat map summarizing the results of feature importance ranking based on 11 feature importance methods. DT: Decision Tree; RF: Random Forest; PC1: first principal component; LOO: Leave-One-Out; PERM: Permutation; RFE_10: Recursive Feature Elimination (RFE) with 10 selected features; RFE_20: RFE with 20 selected features; RFE_30: RFE with 30 selected features; RFE_43: RFE with 43 selected features; Corr: Correlation method.

The visual inspection of the feature distribution indicated the feasibility of using these extracted features for cough detection. As a result, the importance of each individual feature was ranked using the previously discussed feature ranking methods. Some methods, as mentioned earlier, were used multiple times with different parameters. Therefore, a total of 11 feature importance ranking methods were utilized. The features were ranked from 1 to 43, with 1 indicating the highest importance and 43 the lowest. A summary of the ranking is visualized in the heat map shown in Figure 8. The color index represents feature significance, with darker cells indicating higher ranking.

### 3.1 Performance metrics

The feature ranking results for feature selection were evaluated by assessing their performance on the test data set. The performance metrics included accuracy (ACC), sensitivity (SN), specificity (SP), positive predictive value (PPV), negative predictive value (NPV), false positive rate (FPR), false negative rate (FNR), false discovery rate (FDR), and F1 score. These performance metrics are computed as given in Equations 3–11:

(3) $$ACC=\frac{TP+TN}{TP+FP+FN+TN}$$

(4) $$SN=\frac{TP}{TP+FN}$$

(5) $$SP=\frac{TN}{TN+FP}$$

(6) $$PPV=\frac{TP}{TP+FP}$$

(7) $$NPV=\frac{TN}{TN+FN}$$

(8) $$FPR=\frac{FP}{TN+FP}$$

(9) $$FNR=\frac{FN}{TP+FN}$$

(10) $$FDR=\frac{FP}{TP+FP}$$

(11) $$F1=\frac{2(PPV)(SN)}{PPV+SN}=\frac{2TP}{2TP+FP+FN}$$

In these equations, TP (True Positive) represents the number of correctly identified coughs, TN (True Negative) is the number of correctly labeled non-coughs, FP (False Positive) is the number of non-coughs misclassified as coughs, and FP (False Negative) is the number of coughs mislabeled as non-coughs.

### 3.2 Evaluation of feature importance

To evaluate which set of features provided by these methods perform better in distinguishing cough motions, their performance on the classification problem was assessed. The same classifier model, a binary Logistic Regression model, was used for all cases using two evaluation approaches for data splitting into training and testing. In the first performance evaluation approach, the data were split as was shown earlier in Figure 2, using 80% of the records from each subject for training and the remaining 20% for testing. The model was trained on the training dataset using the “top” features chosen by each ranking method. The classifier’s performance was then evaluated on the test dataset using the same set of “top” features selected in the training process. This process was repeated for each of the 11 methods for comparison with the base model, which used all 43 features.

In the second performance evaluation, the model was trained and tested using a leave-one-subject-out (LOSO) data split approach for cross-validation. Using this approach on the collected data from five subjects set the model’s cross-validation folds to 5, where in the i fold, the ith subject was used for testing, while the remaining subjects were used for training. The average results across the five folds were then computed and used for the model’s performance evaluation. Similar to the first approach, this process was also repeated for each of the 11 methods using the “top” features chosen by each ranking method for comparison with the base model using all 43 features.

**Figure 9:**
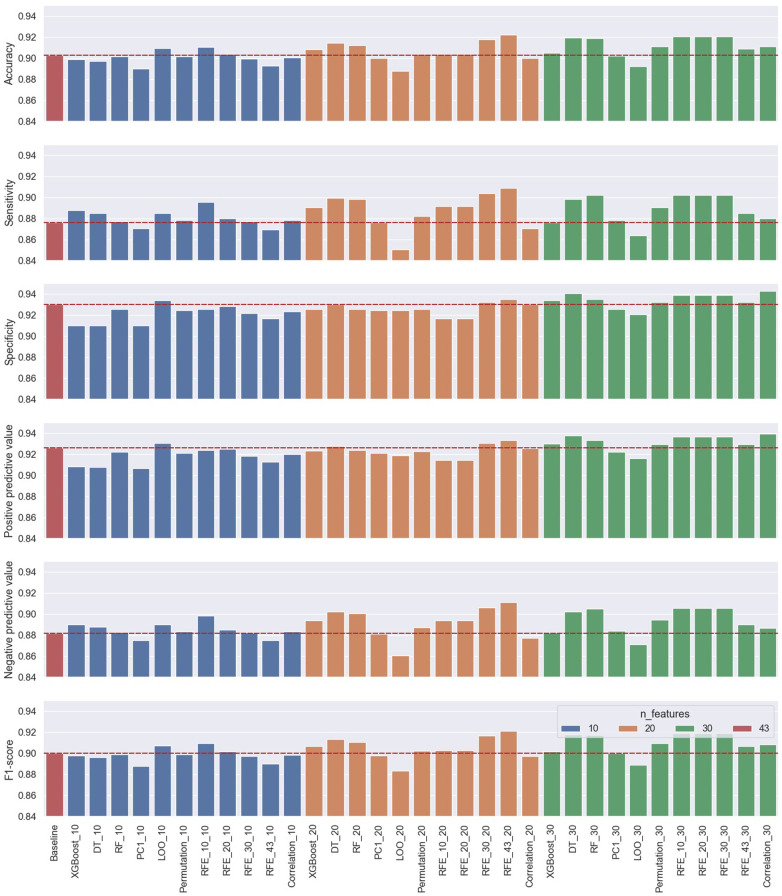
Performance results of the first approach’s 33 classification cases using 10, 20, and 30 selected features from the 11 ranking methods compared to the baseline classification model using all the features.

**Figure 10:**
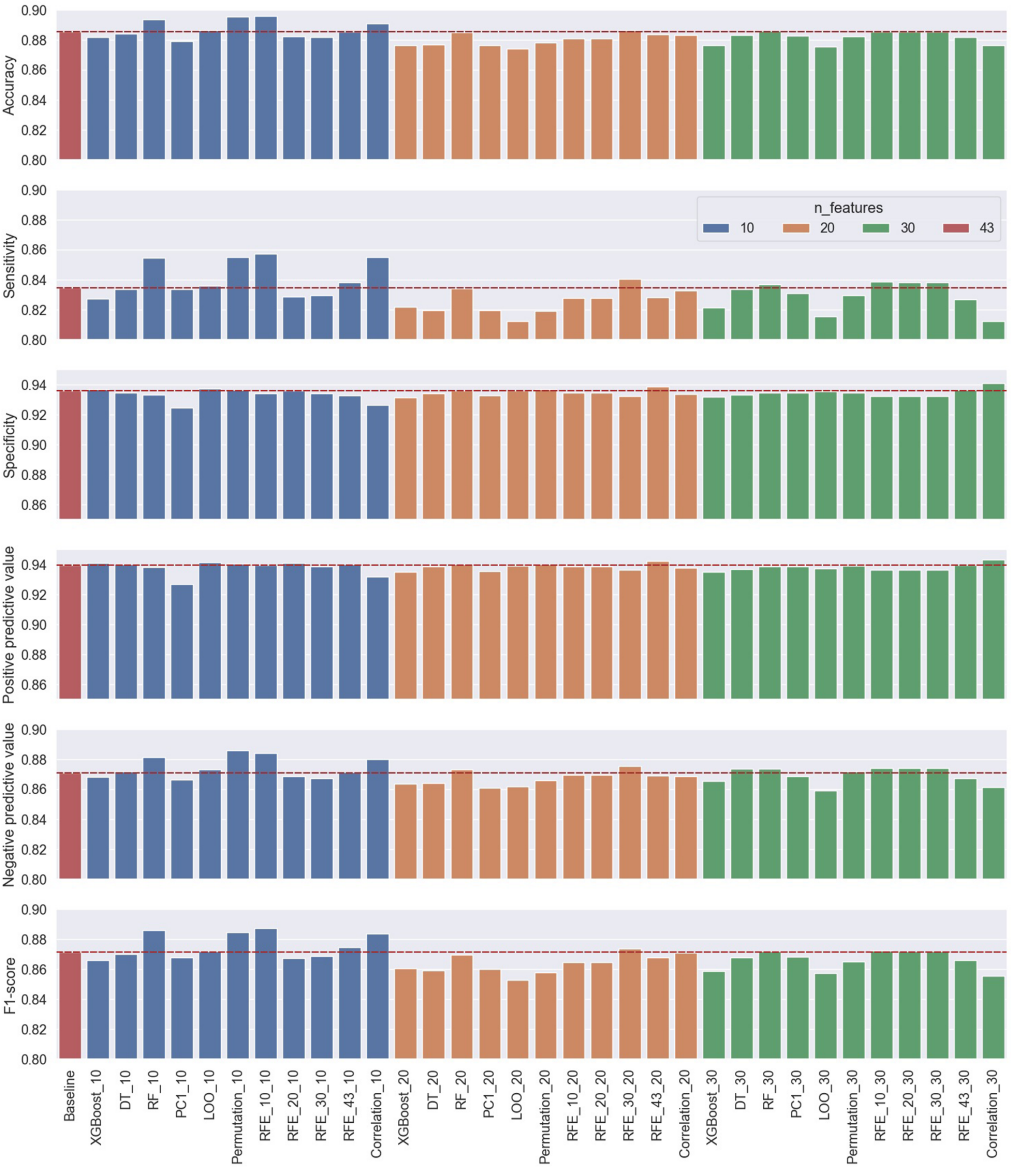
Performance results of the second approach’s (LOSO) 33 classification cases using 10, 20, and 30 selected features from the 11 ranking methods compared to the baseline classification model using all the features.

The logistic regression model was used to also evaluate the trade-off between the number of selected features and model performance on the test data set. The process was repeated for three cases for the number of selected features, using 10, 20, and 30 features, respectively, from each ranking method for both performance evaluation approaches. Therefore, a total of 66 models (33 models/approach) with their performance metrics were evaluated and compared to the baseline model using all features. The results of the performance metrics of all the classification cases using the first approach of subject-record-split are summarized in Table 2 and visually demonstrated in Figure 9 for the six performance metrics. The results of the performance metrics of all the classification cases using the second approach (LOSO) are summarized in Table 3 and Figure 10.

**Table 2:** Summary of classification performance results of the first approach, using features selected by the 11 ranking methods under three cases for the number of features used (n_features = 10, 20, and 30) with the baseline method referring to the use of all 43 features.

Method	ACC	SN	SP	PPV	NPV	F1	FPR	FNR	FDR
Baseline	0.9028	0.8757	0.9299	0.9259	0.8821	0.9001	0.0701	0.1243	0.0741
n_features = 10									
XGBoost	0.8988	0.8876	0.9101	0.9080	0.8900	0.8977	0.0899	0.1124	0.0920
DT	0.8975	0.8849	0.9101	0.9077	0.8877	0.8962	0.0899	0.1151	0.0923
RF	0.9015	0.8770	0.9259	0.9221	0.8827	0.8990	0.0741	0.1230	0.0779
PC1	0.8902	0.8704	0.9101	0.9063	0.8753	0.8880	0.0899	0.1296	0.0937
LOO	0.9094	0.8849	**0.9339**	**0.9305**	0.8903	0.9071	**0.0661**	0.1151	**0.0695**
Permutation	0.9015	0.8783	0.9246	0.9209	0.8837	0.8991	0.0754	0.1217	0.0791
RFE_10	**0.9107**	**0.8955**	0.9259	0.9236	**0.8986**	**0.9093**	0.0741	**0.1045**	0.0764
RFE_20	0.9041	0.8796	0.9286	0.9249	0.8852	0.9017	0.0714	0.1204	0.0751
RFE_30	0.8995	0.8770	0.9220	0.9183	0.8823	0.8972	0.0780	0.1230	0.0817
RFE_43	0.8929	0.8690	0.9167	0.9125	0.8750	0.8902	0.0833	0.1310	0.0875
Correlation	0.9008	0.8783	0.9233	0.9197	0.8835	0.8985	0.0767	0.1217	0.0803
n_features = 20									
XGBoost	0.9081	0.8902	0.9259	0.9232	0.8940	0.9064	0.0741	0.1098	0.0768
DT	0.9147	0.8995	0.9299	0.9277	0.9024	0.9134	0.0701	0.1005	0.0723
RF	0.9120	0.8981	0.9259	0.9238	0.9009	0.9108	0.0741	0.1019	0.0762
PC1	0.9001	0.8757	0.9246	0.9207	0.8815	0.8976	0.0754	0.1243	0.0793
LOO	0.8876	0.8505	0.9246	0.9186	0.8608	0.8832	0.0754	0.1495	0.0814
Permutation	0.9041	0.8823	0.9259	0.9225	0.8872	0.9020	0.0741	0.1177	0.0775
RFE_10	0.9041	0.8915	0.9167	0.9145	0.8942	0.9029	0.0833	0.1085	0.0855
RFE_20	0.9041	0.8915	0.9167	0.9145	0.8942	0.9029	0.0833	0.1085	0.0855
RFE_30	0.9180	0.9034	0.9325	0.9305	0.9062	0.9168	0.0675	0.0966	0.0695
RFE_43	**0.9220**	**0.9087**	**0.9352**	**0.9334**	**0.9111**	**0.9209**	**0.0648**	**0.0913**	**0.0666**
Correlation	0.9001	0.8704	0.9299	0.9255	0.8777	0.8971	0.0701	0.1296	0.0745
n_features = 30									
XGBoost	0.9048	0.8757	0.9339	0.9298	0.8825	0.9019	0.0661	0.1243	0.0702
DT	0.9193	0.8981	0.9405	0.9378	0.9023	0.9176	0.0595	0.1019	0.0622
RF	0.9187	**0.9021**	0.9352	0.9330	0.9052	0.9173	0.0648	0.0979	0.0670
PC1	0.9021	0.8783	0.9259	0.9222	0.8838	0.8997	0.0741	0.1217	0.0778
LOO	0.8922	0.8638	0.9206	0.9158	0.8711	0.8890	0.0794	0.1362	0.0842
Permutation	0.9114	0.8902	0.9325	0.9296	0.8947	0.9095	0.0675	0.1098	0.0704
RFE_10	**0.9206**	**0.9021**	0.9392	0.9368	**0.9056**	**0.9191**	0.0608	**0.0979**	0.0632
RFE_20	**0.9206**	**0.9021**	0.9392	0.9368	**0.9056**	**0.9191**	0.0608	**0.0979**	0.0632
RFE_30	**0.9206**	**0.9021**	0.9392	0.9368	**0.9056**	**0.9191**	0.0608	**0.0979**	0.0632
RFE_43	0.9087	0.8849	0.9325	0.9292	0.8902	0.9065	0.0675	0.1151	0.0708
Correlation	0.9114	0.8796	**0.9431**	**0.9393**	0.8868	0.9085	**0.0569**	0.1204	**0.0607**

The highest scores attained for each case, in all metrics, are emphasized in bold.

**Table 3:** Summary of classification performance results of the second approach (LOSO), using features selected by the 11 ranking methods under three cases for the number of features used (n_features = 10, 20, and 30) with the baseline method referring to the use of all 43 features.

Method	ACC	SN	SP	PPV	NPV	F1	FPR	FNR	FDR
Baseline	0.8852	0.8345	0.9360	0.9394	0.8708	0.8712	0.0640	0.1655	0.0606
n_features = 10									
XGBoost	0.8819	0.8271	0.9367	0.9407	0.8681	0.8658	0.0633	0.1729	0.0593
DT	0.8842	0.8336	0.9348	0.9399	0.8717	0.8701	0.0652	0.1664	0.0601
RF	0.8938	0.8545	0.9331	0.9384	0.8813	0.8857	0.0669	0.1455	0.0616
PC1	0.8792	0.8338	0.9245	0.9266	0.8664	0.8679	0.0755	0.1662	0.0734
LOO	0.8865	0.8357	**0.9374**	**0.9416**	0.8731	0.8719	**0.0626**	0.1643	**0.0584**
Permutation	0.8956	0.8550	0.9362	0.9403	**0.8858**	0.8845	0.0638	0.1450	0.0597
RFE_10	**0.8957**	**0.8571**	0.9343	0.9397	0.8843	**0.8872**	0.0657	**0.1429**	0.0603
RFE_20	0.8823	0.8288	0.9357	0.9408	0.8688	0.8674	0.0643	0.1712	0.0592
RFE_30	0.8819	0.8298	0.9340	0.9386	0.8673	0.8687	0.0660	0.1702	0.0614
RFE_43	0.8854	0.8381	0.9326	0.9398	0.8716	0.8746	0.0674	0.1619	0.0602
Correlation	0.8907	0.8550	0.9264	0.9318	0.8799	0.8835	0.0736	0.1450	0.0682
n_features = 20									
XGBoost	0.8764	0.8217	0.9312	0.9349	0.8634	0.8604	0.0688	0.1783	0.0651
DT	0.8769	0.8195	0.9343	0.9384	0.8642	0.8590	0.0657	0.1805	0.0616
RF	0.8850	0.8340	0.9360	0.9400	0.8732	0.8697	0.0640	0.1660	0.0600
PC1	0.8763	0.8198	0.9329	0.9357	0.8608	0.8601	0.0671	0.1802	0.0643
LOO	0.8740	0.8121	0.9360	0.9391	0.8617	0.8526	0.0640	0.1879	0.0609
Permutation	0.8780	0.8193	0.9367	0.9398	0.8660	0.8579	0.0633	0.1807	0.0602
RFE_10	0.8811	0.8276	0.9345	0.9386	0.8693	0.8646	0.0655	0.1724	0.0614
RFE_20	0.8811	0.8276	0.9345	0.9386	0.8693	0.8646	0.0655	0.1724	0.0614
RFE_30	**0.8864**	**0.8405**	0.9324	0.9364	**0.8755**	**0.8736**	0.0676	**0.1595**	0.0636
RFE_43	0.8835	0.8283	**0.9386**	**0.9422**	0.8690	0.8676	**0.0614**	0.1717	**0.0578**
Correlation	0.8832	0.8329	0.9336	0.9377	0.8687	0.8707	0.0664	0.1671	0.0623
n_features = 30									
XGBoost	0.8765	0.8212	0.9319	0.9349	0.8654	0.8588	0.0681	0.1788	0.0651
DT	0.8833	0.8336	0.9331	0.9368	0.8738	0.8676	0.0669	0.1664	0.0632
RF	**0.8858**	**0.8369**	0.9348	0.9385	0.8737	0.8720	0.0652	0.1631	0.0615
PC1	0.8829	0.8310	0.9348	0.9386	0.8685	0.8684	0.0652	0.1690	0.0614
LOO	0.8752	0.8152	0.9352	0.9374	0.8592	0.8572	0.0648	0.1848	0.0626
Permutation	0.8823	0.8298	0.9348	0.9392	0.8717	0.8649	0.0652	0.1702	0.0608
RFE_10	0.8854	0.8386	0.9321	0.9362	**0.8743**	**0.8721**	0.0679	**0.1614**	0.0638
RFE_20	0.8852	0.8383	0.9321	0.9362	0.8740	0.8719	0.0679	0.1617	0.0638
RFE_30	0.8852	0.8383	0.9321	0.9362	0.8740	0.8719	0.0679	0.1617	0.0638
RFE_43	0.8817	0.8269	0.9364	0.9396	0.8671	0.8658	0.0636	0.1731	0.0604
Correlation	0.8765	0.8124	**0.9407**	**0.9432**	0.8614	0.8555	**0.0593**	0.1876	**0.0568**

The highest scores attained for each case, in all metrics, are emphasized in bold.

## 4 Discussion

In this study, the use of accelerometry signals acquired from a non-contact tri-axial accelerometer sensor was investigated for their ability to differentiate between cough and non-cough events based on motion. This included the extraction of time-domain-related features, the exploration of individual feature distribution based on class, the ranking of the feature’s importance using multiple feature selection methods, and finally the evaluation of classification performance based on the number of selected features chosen by each ranking method using two data splitting approaches: subject-record-split and LOSO.

The visualization of feature distribution using the box plots given in Figures 4–7 provided a primary insight into the feasibility of using detected motion to distinguish cough events. The 11 features extracted from the accX given in Figure 4 presented distributions where the majority of the features have different median, upper quartile, and lower quartile values between both classes of cough and non-cough. This observation indicated that at least 50% of the data points of a particular feature have distinct values for each class, such as Min_X, and Diff_X. However, there are three features (Skewness_X, Kurtosis_X, and Ent_X), which presented an overlap in their distribution, where though they had different median values for each class, their lower and upper quartile values overlap. In this case, differentiating between cough and non-cough events by simply depending on these features alone would be tricky.

Similar observations were made for the data distributions of features extracted from accY, accZ, and Mag signals. In the case of accY features given in Figure 5 and accZ features in Figure 6, 8 out of the 11 features of each axis have distinct values for the median and quartiles of each class, indicating its possible use in identifying cough motions. The three remaining features of each axis presented with overlapped distribution between cough and non-cough events. These features were the skewness, kurtosis, and correlation features (Skewness_Y, Kurtosis_Y, Corr_yz, Skewness_Z, Kurtosis_Z, and Corr_xz). Nevertheless, they can still be helpful if used together with other features, as they all had different median values for each class. Especially in the case of Corr_xz, where there is an overlap, it is only with one quartile—the upper quartile of the cough data with the lower quartile of the non-cough data points. As for the features extracted from the Mag signal in Figure 7, they share the same trend as the previous axes, where 8 out of the 10 features have distinct median, lower quartile, and upper quartile values for each class, while the two features related to skewness and kurtosis presented distinct median values with overlap in quartile values. However, it is noticed that the distribution of data points for the features extracted from the magnitude signals has a more distinctive distribution separating the two classes when compared to the features extracted from the other axes. As observed in the following features (Min_Mag, Max_Mag, Diff_Mag, RMS_Mag, Var_Mag, and Ent_Mag), the full range of the non-cough data points overlap only with part of the whisker of the cough data points, indicating an overlap that is less than 25%. This was also observed in 4 features extracted from the accZ (Min_Z, Max_Z, Diff_Z, and RMS_Z), reflecting these 10 features’ higher potential in distinguishing cough events. As for the presence of outliers, it was expected as data were collected from a total of five subjects, where it is highly unlikely to have exactly similar coughing motions from all subjects of different genders, hence the presence of outliers.

A qualitative analysis of the extracted features was implemented using the 11 feature ranking methods to evaluate each feature’s importance toward cough identification. It is noticed from the heat map in Figure 8—summarizing the ranking of all features based on method—that features extracted from the magnitude signal rank higher than other features. The Diff_Mag ranked first by four methods and ranked second by another two methods. Min_Mag also had one of the top rankings showing a fully dark row across all methods similar to Diff_Mag. Other features that ranked first were the Max_Z by the RFE_10 and RFE_20 methods as well as the Corr_xy by permutation and the Corr_xz by LOO method. Corr_xy also ranked second by four other methods (XGBoost, DT, RFE_10, and RFE_20) reflecting its importance, where half of the methods ranked it in the top two features. As for the least important features, it is perceived that the upper half of the heat map, relating to features extracted from the accX and some of the accY features, are lighter in color with a ranking above 20. Still, there are some anomalies in this observation, such as the rank 3, 5, and 6 for RMS_X by XGBoost, DT, and permutation methods, respectively, as well as the first rank given to Min_X by both methods. A simplified summary of the ranking results is given in Table 4, where the feature ranking is set to three groups indicating the rank in the top 10 (rank 1–10), top 20 (rank 11–20), or top 30 (rank 21–30). This summary focuses more on the presence of a feature in a top category rather than its specific ranking, which helps simplify the analysis and comparison of the feature rankings by the different methods. For example, it is noticed that all ranking methods place Min_Mag in the top 10 set of features; thus, from this observation, it can be assumed that Min_Mag is the most important feature.

**Table 4:** Summary of features used by each ranking method grouped by their ranking into three categories: Top 10 (∘), Top 20 (□), and Top 30 (△), where empty cells indicate a ranking greater than 30.

Feature	XGBoost	DT	RF	PC1	LOO	PERM	RFE_10	RFE_20	RFE_30	RFE_43	Corr
Min_X	△	△		△	∘	□	△	△	∘	∘	△
Max_X	□		□	△	∘		△	△	△	△	□
Diff_X	△	△	△	△	△	□	△	△	△	△	□
RMS_X	∘	∘	△		□	∘	□	□	△	△	△
Skew_X	□	□		□	□					△	
Kurt_X	△	△		∘	△	∘				△	
IQR_X		△			△	□				△	
Var_X			△		□		□	□	△	△	△
Ent_X	□	△	△	△			△	△	△		
MAD_X					△	△					△
Min_Y		△		△		□					□
Max_Y	□	□	□	□		∘	△	△	△		□
Diff_Y	□	□	□	□		□	□	□	△		□
RMS_Y	∘	□	□		△	△	△	△	△		△
Skew_Y				∘	△	△					
Kurt_Y		□		∘	□						
IQR_Y					□						△
Var_Y			□			△	△	△	△		△
Ent_Y	△	□	△	□	∘		□	□	△		
MAD_Y	△		△		∘					△	△
Min_Z		△	∘	□		□	□	□	□		∘
Max_Z	□	□	∘	□	□	□	∘	∘	□	△	∘
Diff_Z	∘	∘	∘	∘		∘	∘	□	∘	∘	∘
RMS_Z	∘	△	□	△	□	△	□	□	∘	∘	∘
Skew_Z	□	□		△			△	△	∘	∘	
Kurt_Z				∘	□					∘	
IQR_Z	△									∘	□
Var_Z		□	□			△	∘	□	∘	∘	□
Ent_Z	∘	∘	□	□	△	□	□	∘	∘	∘	△
MAD_Z			△		∘					∘	□
Min_Mag	∘	∘	∘	∘	∘	∘	∘	∘	∘	∘	∘
Max_Mag	△		∘	∘	∘	∘	∘	∘	∘	□	∘
Diff_Mag	∘	∘	∘	∘	∘	∘	∘	∘	∘	□	∘
RMS_Mag	∘		∘	□			∘	∘	∘	□	∘
Skew_Mag	△	△	△	∘	△	△	△	△	□	□	
Kurt_Mag	□	∘	△	∘	□	△	□	∘	□	□	
IQR_Mag	△		△		□	∘	△	△	□	□	□
Var_Mag		∘	∘	△		□	∘	∘	□	□	∘
Ent_Mag	△	△	∘	□	△	□	□	∘	□	□	∘
MAD_Mag	□	□				△				□	□
Corr_xy	∘	∘	∘	△	∘	∘	∘	∘	□	□	△
Corr_yz	□	∘	□	△	△	△	□	□	□	△	
Corr_xz	∘	∘	□	□	∘	∘	∘	□	□		

Overall, the feature importance methods resulted in different rankings across the features. This is expected as the ranking methods measure feature importance in different ways. Model-based methods (XGBoost, DT, and RF) depend on the built-in importance of measuring the decrease in impurity. They have different biases than other methods, where tree-based models favor features that minimize the number of splits. While the PCA method relies on the cumulative explained variance of the first principal component and its correlation to the features. The LOO, permutation, and RFE methods measure the importance based on the drop in the prediction accuracy when a feature is removed or shuffled. Each of these three methods uses a different feature elimination approach, hence the difference in ranking results. As for the correlation method, it measures the direct correlation between each feature and the expected output class.

Another observation is the relatively close ranking results produced by some methods, such as RFE_30 and RFE_43. An explanation behind this ranking overlap is the algorithm behind this methodology to rank the features. Both are based on the recursive feature elimination (RFE) method, but the first was set for 30 selected features, while the other was set for 43 features. The ranking behind this method relies on recursively searching the feature pool to select the best n features and then prune out the remaining less important features. Thus, when the difference is only the number of selected features, it is likely to have some overlap in the ranking results, which is observed in the results of the four methods based on RFE with n=10, 20, 30, and 43. This trend was also observed by the three tree-based ranking methods—XGBoost, DT, and RF—where the results do not completely overlap but are very close to each other due to some similarities in these models’ architectures.

The performance results of the first approach, summarized in Figure 9 and Table 2 using the subject-record-split, show that the baseline model using all 43 features had already presented good performance. However, as one of the objectives of this study is to assess and examine the trade-off between performance and the number of features used, our target is to find optimum models presenting similar or improved performance to the baseline using a lower number of features. Examining the use of only 10 features, it is noticed that three models outperformed the baseline in terms of accuracy with the best score of 0.9107 achieved by the RFE_10 model. In terms of sensitivity, 9 out of the 11 models achieved better scores with the best being 0.8955 by the RFE_10 model. As for specificity and PPV, the LOO model was the only one that performed better than the baseline with 0.9339 for specificity and 0.9305 for PPV. The NPV improved in eight models while staying the same for the RFE_30 model, with the highest NPV at 0.8986 again by the RFE_10 model, which also reported the highest F1 score at 0.9093. As for the three remaining metrics, the best scores of FPR and FDR were achieved by the LOO model at 0.0661 and 0.0695, respectively, while the best FNR was 0.1045 achieved by the RFE_10 model. Since FPR is complementary to specificity, FNR to sensitivity, and FDR to PPV, only the first six metrics are used for comparison in the rest of the discussion.

When 20 features were selected, the RFE_43 model presented the best performance across all metrics, outperforming the baseline model. It increased the baseline accuracy by 0.0192, sensitivity by 0.33, specificity by 0.0053, PPV by 0.0075, NPV by 0.29, and F1 score by 0.208. Overall, the use of 20 features, in comparison to 10 features or the baseline, in the majority of the models improved the classification accuracy, sensitivity, NPV, and F1 score; having at least 8 out of the 11 models with scores above the baseline. As for the specificity and PPV, only two and three models, respectively, scored slightly higher than baseline.

Regarding the use of 30 features for the classification problem, the performance metrics of three methods, namely, RFE_10, RFE_20, and RFE_30, achieved the best accuracy, sensitivity, NPV, and F1 scores, while the correlation model achieved the best specificity and PPV scores. It is also noticed that the three models RFE_10, RFE_20, and RFE_30 achieved the same performance, which leads back to the point discussed earlier, about the close ranking of these methods—where the top 30 features (regardless of rank order) of these models were the same. This can be easily visualized in the summarized Table 4.

As a measure of the generalizability of the proposed cough detection method, the LOSO approach was also used for evaluation. The results of this approach summarized in Figure 10 and Table 3 reflected an acceptable performance from the baseline using all 43 features. Although these baseline results were different than those reported by the subject-record-split approach, this was expected as the model was tested on unseen subjects and the results did not show major deviation. A slight drop in accuracy (−0.0176), sensitivity (−0.0412), NPV (−0.0112), and F1 score (−0.0289) were noticed while an improvement in specificity (+0.0061) and PPV (+0.0135) in the LOSO baseline model were noticed. In this approach, it was observed that the increase in the number of features from 10 to 20 to 30 did not necessarily improve the model’s performance. In terms of accuracy, sensitivity, NPV, and F1 score, 6 out of 11 models using only 10 features resulted in notably improved performance, while only 1 model with 20 features and 4 models with 30 features resulted in a slightly improved performance. As for specificity, three models with 10 features, four models with 20 features, and two models with 30 features reported improved performance. The reported PPV scores noted an improvement in seven models with 10 features, three models with 20 features, and only two models with 30 features. It is important to note that the increase in performance scores of both specificity and PPV were borderline as noticed in Figure 10, hence they did not affect the choice of the best-performing model. The best-performing model out of the 33 models in this approach was the RFE_10 model using only 10 features reporting the best accuracy, sensitivity, and F1 score with an increase from the baseline score by 0.0105, 0.0226, and 0.0160, respectively.

The general observation of the models’ performance shown in Figures 9 and 10 present the following trend, where accuracy, sensitivity, NPV, and F1 score have similar behavior as the number of features increases with dependency on the model used, while specificity and PPV share a closer behavior—both metrics that consider false positives (FP) in their computation. In the subject-record-split approach of Figure 9, it can be deduced that only a few models using 10 or 20 features were able to improve the model’s performance in terms of lowering FPs, while when the number of features was increased to 30, the majority of the models (9 out of 11) achieved higher specificity and PPV scores than the baseline. As for the LOSO approach, it is noticed that more models using 10 features were able to achieve a slightly better performance than the baseline, and as the number of features increased, less number of models were able to improve the performance reaching only two models using 30 features, one of which achieved the best specificity and PPV across all 33 models. As for the four other metrics, they were able to achieve maximum improvement using only 20 features in the subject-record-split approach and only 10 features in the LOSO approach. Therefore, we can conclude that it is possible to use as low as 10 features for cough detection following the results presented by the LOSO approach of model RFE_10 to achieve an accuracy of 0.8957, sensitivity of 0.8571, specificity of 0.9343, PPV of 0.9397, NPV of 0.8843, and F1 score of 0.8872. We can also use up to 20 features based on the results of the subject-record-split best-performing model, the RFE_43 with an accuracy of 0.9220, sensitivity of 0.9087, specificity of 0.9357, PPV, of 0.9334, NPV of 0.9111, and F1 score of 0.9209. In both cases, though the improvement from the baseline model is relatively small, it is however achieved with less than a half (subject-record-split) and/or a quarter (LOSO) of the total number of features, which will eventually reflect on the size of the model, computational time, and space required.

Examining the list of features selected by the best-performing models using the summary in Table 4, it is noticed that eight features are common between the best-performing models of both approaches. In the LOSO approach using the top 10 features, 5 features were related to the Mag signal, 3 to the accZ signal, and 2 to the correlation between axes Corr_xy and Corr_xz. As for the subject-record-split approach using the top 20 features, 10 features were extracted from the magnitude signal, 8 features from the accZ signal, and Corr_xy and Min_X features. Among these features, the Min_Mag can be considered the top feature as it was ranked in the top 10 by all 11 methods, even though none ranked it as 1. It is followed by Diff_Mag, which was ranked in the top 10 by 10 methods, and Diff_Z, which was ranked in the top 10 by 9 methods, and then Max_Mag and Corr_xy. These five top features are common between the two best-performing models. As for features that ranked as least important, IQR_Y was only ranked by two methods, once in the top 20 and another in the top 30, while the remaining 9 methods ranked it above 30. Similarly, MAD_X, Skew_Y, Kurt_Y, Kurt_Z, and IQR_Z, were only ranked by three methods to be within the top 30 features. Nevertheless, within these three ranking methods, some ranked within the top 10 features, specifically the accZ features, which were both used in the best-performing model. Therefore, it is not enough to only evaluate the ranking of features individually, but rather as a set used for the classification problem.

As this study investigates a relatively new approach to cough detection that has not been explored before, the results of our early-stage investigation have some limitations that need to be addressed in future work. One of the main limitations is related to the used dataset, where the data were collected from only five subjects. Another is that the collected data represent cough and non-cough events only when the subjects are seated. These limitations highlight the need for subsequent work to further collect data from a wider population and under different states such as walking and/or lying down. In addition, as there were limited data available, our initial evaluation used a subject-record-split approach for data splitting. This facilitated the performance evaluation of the cough detection models using unseen records from trained subjects for testing the models; this reported good performance but questioned the generalizability of the models on unseen subjects. To overcome this issue, the second approach using LOSO was implemented and proved the ability of the proposed cough detection solution to perform well on unseen subjects. However, the results from the LOSO approach can be considered a bit biased as one out of the five subjects had half the number of total records compared to the other four subjects, resulting in better performance during its testing fold. Nevertheless, combining the results from both approaches can compensate for the limitations of individual approaches.

In conclusion, this study presented an overall evaluation of different extracted features for motion-based cough detection. It demonstrated the ability to use as minimum as 10 time-domain features, extracted from the acquired signals of the non-contact accelerometer, to distinguish between cough and non-cough events and reflected the redundancy of some features. It presented 68 different classification models using two evaluation approaches for data splitting, based on 11 different feature ranking methods and 3 sets of number of selected features, which can be used and modified for future improvement. It also paves the way for further development, allowing the use of more sophisticated classifiers and expanding on the type of detected motions such as walking and standing. Overall, this evaluation study serves the main purpose of presenting a guide for future research related to the use of motion-based cough detection.

## Data Availability

The raw data supporting the conclusions of this article will be made available by the authors, without undue reservation.
